# Supplementary figures and images for: Wastewater surveillance of SARS-CoV-2 genomic populations on a country-wide scale through targeted sequencing
Source: PLoS One. 2023 Apr 21;18(4):e0284483. doi: 10.1371/journal.pone.0284483 (PMC10121012; doi:10.1371/journal.pone.0284483)

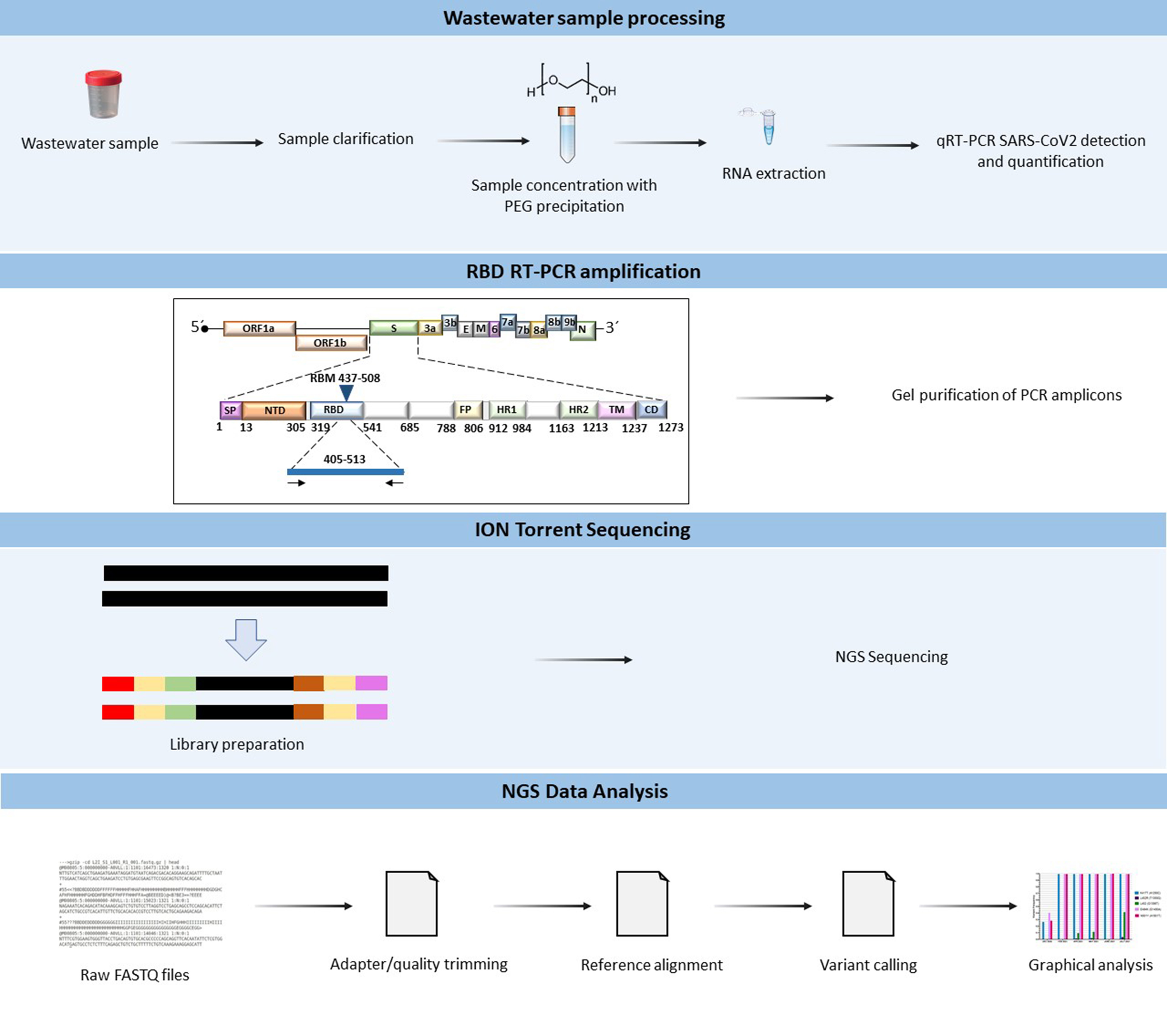

Supplement: S1 Fig — (TIF) [file pone.0284483.s001.tif]
