# Supplementary material for: Wastewater surveillance of SARS-CoV-2 genomic populations on a country-wide scale through targeted sequencing
Source: PLoS One. 2023 Apr 21;18(4):e0284483. doi: 10.1371/journal.pone.0284483 (PMC10121012; doi:10.1371/journal.pone.0284483)
Supplement: S1 Table — (DOCX) [file pone.0284483.s002.docx]

**Table S1.** Real-time PCR Ct value, date and sampling place from Uruguayan

waste-water samples.

| Sample Number | Ct Value | Date | Place |
| --- | --- | --- | --- |
| 13 | 27.05 | Dec 2020 | Rivera |
| 14 | 28.50 | Feb 2021 | Rivera |
| 15 | 27.00 | Apr 2021 | Rivera |
| 16 | 28.58 | May 2021 | Rivera |
| 17 | 28.91 | June 2021 | Rivera |
| 45 | 27.07 | June 2021 | Melo |
| 18 | 29.88 | July 2021 | Rivera |
| 0 | 32.47 | July 2021 | Montevideo |
| 7 | 28.73 | July 2021 | Castillos |
| 10 | 28.12 | July 2021 | Salto |
| 12 | 29.14 | July 2021 | Melo |
