# Supplementary material for: Wastewater surveillance of SARS-CoV-2 genomic populations on a country-wide scale through targeted sequencing
Source: PLoS One. 2023 Apr 21;18(4):e0284483. doi: 10.1371/journal.pone.0284483 (PMC10121012; doi:10.1371/journal.pone.0284483)
Supplement: Attachment — Submitted filename: Response to reviewers.docx [file pone.0284483.s003.docx]

To whom it may concern,

Thanks for your comments and suggestions. Please find below a detailed response to your points.

1. Please provide additional details regarding participant consent. In the Methods section, please ensure that you have specified (1) whether consent was informed and (2) what type you obtained (for instance, written or verbal). If your study included minors, state whether you obtained consent from parents or guardians. If the need for consent was waived by the ethics committee, please include this information.

*None of our studies included human subjects. The only samples obtained by the authors were wastewater samples. All the clinical data obtained was in an aggregated format from public dashboards. We have included a sentence regarding this issue in the Methods section of the paper*.

2. Please amend your current ethics statement to include the full name of the ethics committee/institutional review board(s) that approved your specific study.

N/A

3. Firstly, please note that PLOS ONE is unable to publish previously copyrighted maps or satellite images, or images created using proprietary data. Unfortunately, we are unable to confirm if the map in Figure 2 with your submission from Instituto Nacional de Estadística (INE), [www.ine.gub.uy](http://www.ine.gub.uy/), has been provided with licensing that qualifies for publication in PLOS. In order to use these images in your submission, we require explicit permission from the copyright owner to publish the figures under the CC BY 4.0 license.

*We have removed the original map. The Population Density per department indicated in the map is from the Anuario Estadistico Nacional 2019, 96^a^ version, Instituto Nacional de Estadística (INE), www.ine.gub.u y. The map of Uruguay was obtained from USGS National Map Viewer (*[*http://viewer.nationalmap.gov/viewer/*](http://viewer.nationalmap.gov/viewer/)*), one of the resources included in your list.*

Secondly, before we can proceed with your manuscript, we ask that you please clarify the following:

a) Please explain where the authors obtained the images in "SFigure1.tif" in your submission or if the authors created the images themselves.


*All images now were created by the authors.*

**Previous response to editor and reviewers**

To whom it may concern,

1- We have introduced needed changes in formatting to meet PLOS ONE's style requirements.

*The size of the titles for each section have been updated and the files have been labeled correctly.*

2- We have described the role of the funders:

*Funding was provided by the National Center for Science and Civic Engagement to DSS and MT to support wastewater research in the global south. The grant supported the costs associated with the RNA extraction and sequencing. The funders had no role in study design, data collection and analysis, decision to publish, or preparation of the manuscript.*

3- We have identified the source of Figure 2. It is included in the Anuario Estadistico Nacional 2019 96^a^ version. In this publication it is stated that the work can be partially or completely reproduced for free if the Instituto Nacional de Estadística (INE), [www.ine.gub.uy](http://www.ine.gub.uy), the source, is mentioned. (Spanish version on the original document:

Copyright ©Instituto Nacional de Estadística, 2019. Esta publicación debe citarse como “Anuario Estadístico Nacional 2019, 96ª versión”).

La autorización para reproducir total o parcialmente esta obra es libre mencionando la fuente, Instituto Nacional de Estadística (INE), [www.ine.gub.uy](http://www.ine.gub.uy)).

*We have included the source on the Figure 2 caption*.

4. Please include captions for your Supporting Information files at the end of your manuscript, and update any in-text citations to match accordingly.

*We have included the captions for the Supporting Information files at the end of the manuscript an updated in-text citations.*

5- Wastewater surveillance was found to be a powerful and complementary approach to clinical testing in monitoring the SARS-CoV-2 pandemic. Authors report surveillance of variants of concern (VOCs) or No-VOCs agrees with clinical testing. It would be interesting to correlate these temporal changes with clinical data or the confirmed cases with statistical comparisons.

*We have reviewed the literature and the publicly available epidemiological reports and research papers only report changes over time of VOCs (e.g gamma, delta). We have included those references in the paper.* *There are no references to amino acid changes in clinical cases.*

6 - Figure 2 amino acids and their frequencies are shown in circular graphs for each location. Is this a Pie chart? Presented in the form of a Pie should sum to 100%.

*We have changed the representation of amino acids references in Figure 2.*

7- The scale on the Y-axis for Figure 1C is not completely visible

*Figure 1C has been modified to make the scale on the Y-axis completely visible.*

8- It is somewhat surprising to see OK091006 used as a reference. While OK091006 indeed represents an example of the Delta variant of SARS-CoV-2 it is a Genbank record without extensive information on its provenance and quality control. The authors do not explain why this was chosen in addition to the more conventional Wuhan Hu-1 reference (which has been the subject of several revisions and has been deposited in NCBI's RefSeq collection of curated sequences)

*We used the Wuhan Hu-1 and the Delta variant,OK091006, and similar results were obtained for both analyses. The OK091006, sequence was used because it has the same original variations as the Wuhan sequence (MN908947) and it also presents mutations associated with Delta (L452R, T478K), within at least the RBD region of the delta B.1.617.2 lineage.*

9- The authors claim that data is available in NCBI SRA but I could not find it using the accession numbers provided. If the data is embargoed pending publication the authors should state that.

*The data is embargoed pending publication. We have added this statement to the manuscript.*
